# Supplementary material for: Frustration-driven C4 symmetric order in a naturally-heterostructured superconductor Sr2VO3FeAs
Source: Nat Commun. 2017 Dec 18;8:2167. doi: 10.1038/s41467-017-02327-0 (PMC5735138; doi:10.1038/s41467-017-02327-0)
Supplement: Supplementary file 1 — Supplementary Information [file 41467_2017_2327_MOESM1_ESM.pdf]

Supplementary Note 1. X-ray diffraction on Sr_2VO_3FeAs single crystals

Supplementary Fig. 1 shows X-ray diffraction patterns from a Sr_2VO_3FeAs single crystal at various (hkl) planes. The clear spots with no splitting confirm good crystallinity of the crystals without twin domains. The structure information determined from the single crystal XRD was listed in Supplementary Table 1. We note that the tetragonal crystal symmetry ($P4/nmm$) describes well the observed Bragg reflections both at 297 K ($> T_0$) and at 93 K ($< T_0$). A refined crystal structure of Sr_2VO_3FeAs is shown in Supplementary Fig. 1n.

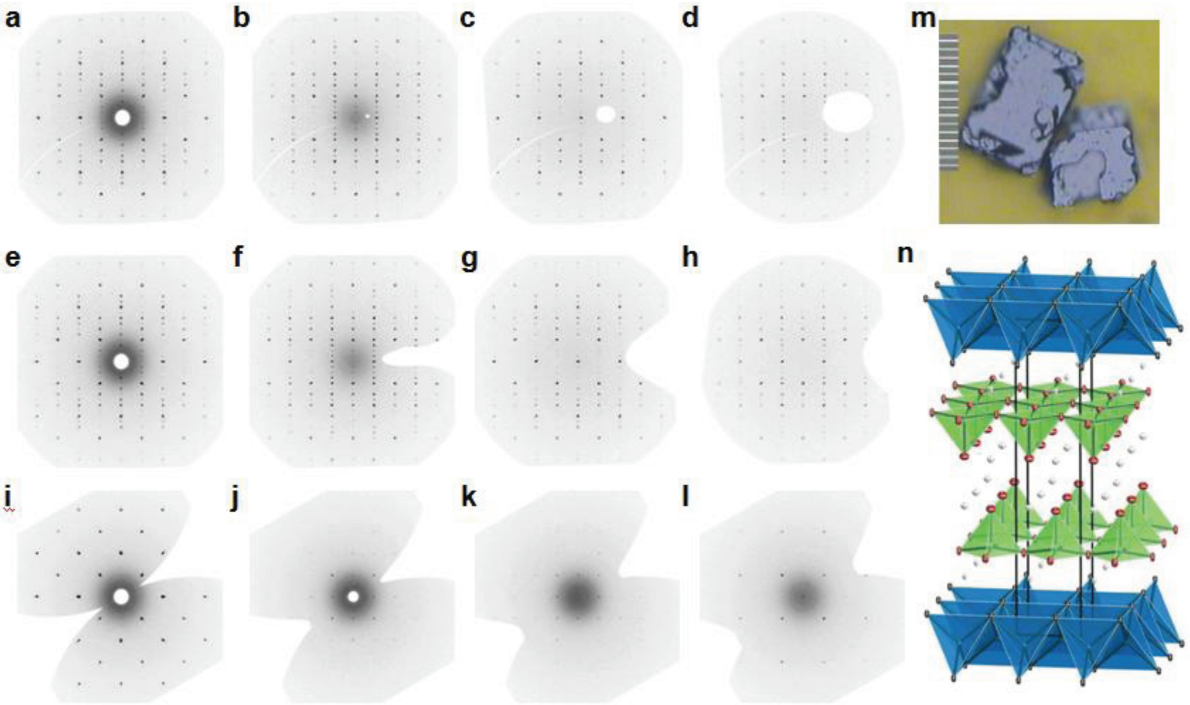

Supplementary Fig. 1: Single crystal X-ray diffraction patterns of Sr_2VO_3FeAs for **a** ($0kl$) **b** ($1kl$) **c** ($2kl$) **d** ($3kl$) **e** ($h0l$) **f** ($h1l$) **g** ($h2l$) **h** ($h3l$) **i** ($hk0$) **j** ($hk1$) **k** ($hk2$) **l** ($hk3$) planes. **m** A photograph of Sr_2VO_3FeAs single crystal grown by the self-flux method (scale bar size is 200μm). **n** Perspective view of the unit cell of Sr_2VO_3FeAs (light gray: Sr, blue: Fe, green: V, red: O, dark gray: As). All ellipsoids are drawn at a 90 % probability level. The square pyramids in green show the coordination of V(1) by four O(1) and one O(2), the blue tetrahedra depict the coordination of Fe(1) by four As(1). The c vector is vertical.

Supplementary Fig. 2a shows the single crystal X-ray diffractograms for two Bragg reflections at (h k l) = (0 0 10) and (2 0 0) as a function of temperature. A weak but clear kink at $T_0 = 155$ K is observed for the (0 0 10) reflection, while no noticeable anomaly appears for the (2 0 0) reflection. Accordingly, as shown in the Supplementary Fig. 2b, the temperature dependence of the c-axis lattice parameter (c) exhibits an anomaly at $T_0 = 155$ K, whereas the a-axis lattice parameter (a) changes monotonically across T_0. This is consistent with the previous reports [1–3] and also the refinement result on single crystal X-ray diffraction data, taken at 93 K ($< T_0$). Therefore, the tetragonal symmetry of Sr_2VO_3FeAs remains unchanged below T_0 within experimental resolution. For other iron pnictides showing an orthorhombic distortion, the orthorhombicity $\delta = (a-b)/(a+b)$ is typically $0.5-4\times10^{-3}$ [4–7]. Considering that the beam divergence of our single crystal X-ray diffractometer is less than ≈ 0.1 degree, the orthorhombicity, if any, should be less than $\delta \sim 10^{-4}$ in Sr_2VO_3FeAs, at least one order of magnitude smaller than found in other iron pnictides. These observations suggest that the nematic C_2 order is either absent or strongly suppressed in Sr_2VO_3FeAs, highly distinct from other iron pnictides. One can argue that the coupling with the $SrVO_3$ layers hardens the Fe lattice. Indeed our density functional theory (DFT) calculations for a putative stripe order produced an optimized structure with $b/a-1 \lesssim 0.5\%$, while similar calculations for $BaFe_2As_2$ yield 1.5%. Still, it is factor of three short of the observed order-of-magnitude difference.

On the contrary, the c-axis lattice parameter suddenly decreases at T_0 by $\Delta c \approx -0.03$ Å. The reduction is comparable with the orthorhombic distortion $a-b \approx 0.02$ Å in other iron pnictides showing a C_2 structural transition. As discussed in the main text, we attribute this reduction of the c-axis lattice parameter to the anion-height change due to an

Supplementary Table 1: Selected crystallographic information from the structure refinement of the XRD patterns of a Sr_2VO_3FeAs single crystal. The standard deviations of the last digit are given in parentheses. The R values for the Rietveld refinement are $R_{int} = 0.0931$, $R1 = 0.0512$, and $wR2 = 0.0671$.

Data collection temperature [K]		293(2)	93(2)
Space group		$P4/nmm$	$P4/nmm$
Formula units per unit cell	Z	2	2
Lattice parameters [Å]	a	3.9155(7)	3.9105(6)
	c	15.608(4)	15.5077(3)
Unit cell volume [Å^3]	V	239.29(9)	237.14(1)
Calculated density [g · cm^3]	ρ	5.620	5.653
Radiation, wavelength [Å]	λ	Ag-K, 0.56083	
Monochromator		curved graphite single crystal	
Atomic parameters			
Sr(1)	[2c (1/4, 1/4, z)]	0.58642(9)	0.58690(8)
Sr(2)	[2c (1/4, 1/4, z)]	0.81000(8)	0.81081(7)
V(1)	[2c (1/4, 1/4, z)]	0.30763(18)	0.30648(15)
O(1)	[4f (3/4, 1/4, z)]	0.2935(5)	0.2926(7)
O(2)	[2c (1/4, 1/4, z)]	0.4291(7)	0.4301(10)
Fe(1)	[2a (3/4, 1/4, z)]	0	0
As(1)	[2c (1/4, 1/4, z)]	0.08954(10)	0.08941(9)

C_4-symmetry vestigial charge or orbital ordering as discussed in the main text. Our DFT calculation have shown, for instance, that the "double-Q" configuration with half Fe atoms magnetic converges to a structure with the lattice parameter c roughly 0.03 Å lower than in fully magnetic stripe phase.

Supplementary Note 2. Magnetic properties of Sr_2VO_3FeAs single crystals

Above T_0, the magnetic susceptibility follows the Curie-Weiss law expressed by $\chi(T) = C/(T - T_{CW}) + \chi_0$ as shown in the Supplementary Fig. 3a. Here T_{CW} is the Curie-Weiss temperature, and χ_0 is the contribution from itinerant Fe electrons, which in other iron pnictides exhibits a temperature-independent or weakly-dependent behavior[8–10]. C is

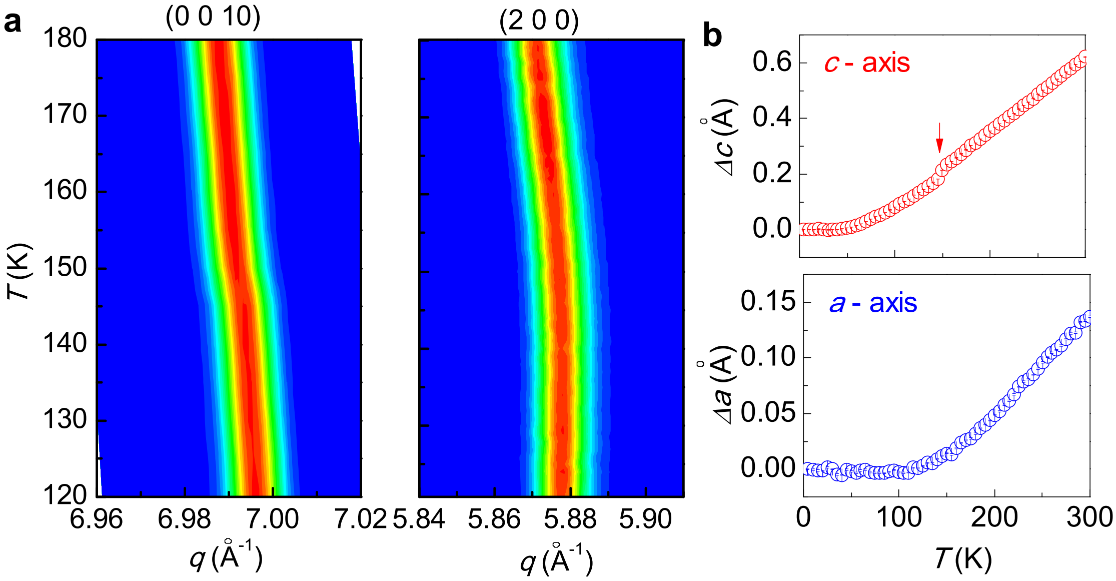

Supplementary Fig. 2: **a** Single crystal X-ray diffraction data from the (0 0 12) and (2 0 0) Bragg peaks as function of temperature. **b** Temperature dependence of the relative change in the a- and c-axis lattice parameters. An anomaly in the c-parameter at T_0 is marked by the arrow.

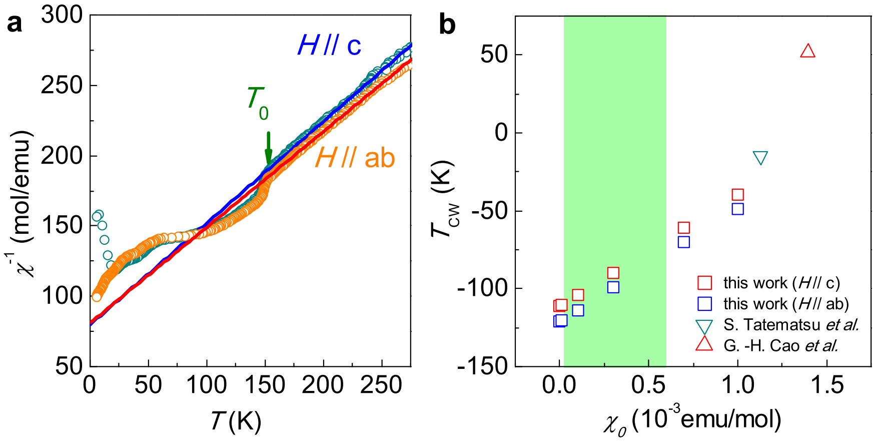

Supplementary Fig. 3: **a** The inverse magnetic susceptibility, $\chi^{-1}(T)$, as a function of temperature for $H||c$ and $H||ab$. The hidden order transition at T_0 is indicated by the arrow. The solid lines are the fit to the Curie-Weiss law. **b** The estimated Curie-Weiss temperature T_{CW} with variation of χ_0. The typical χ_0 value for doped iron pnictides is indicated by green shadow.

the molar Curie constant, $C = (NP_{eff}^2)/3k_B$, with the Avogadro's number N and molar effective magnetic moment P_{eff}. The magnitude of χ_0 is typically $\sim 10^{-4}$ emu/mol, smaller by two orders of magnitude than the observed $\chi(T)$ in Supplementary Fig. 3a. Assuming negligible χ_0, we can fit the experimental data well enough, yielding the Curie-Weiss temperature $T_{CW} = -110(1)$ K for $H \parallel c$ and $T_{CW} = -119(2)$ K for $H \parallel ab$. The estimated effective magnetic moments $P_{eff} = (3k_BC/N)^{1/2}$ from the fit are 2.35 μ_B/V for $H||c$ and 2.43 μ_B/V for $H \parallel ab$. These values are comparable with the expected value $P_{eff} = g\sqrt{S(S+1)}\mu_B = 2.83\mu_B$ for $S = 1$ spins in V^{3+} ions of the $SrVO_3$ layer. The slight reduction of the measured P_{eff} can be attributed to hybridization.

The Curie-Weiss behavior from the localized V spins dominates the temperature dependence of $\chi(T)$. For typical doped iron pnictides without a long-range order [8–11] χ is less than $\sim 0.7 \times 10^{-3}$ emu/mol and very weakly $T-$dependent. We tried several value of χ_0 within this range and obtained the T_{CW} values of -100 ± 30 K, as shown in Supplementary Fig. 3b. For comparison we also plot the T_{CW} estimates in previous reports [1, 2]. Note that the studies reporting a nearly zero or positive T_{CW} utilized $\chi_0 \approx 1.2 - 2.0 \times 10^{-3}$ emu/mol, much larger than typical values for iron pnictides. Setting χ_0 as a fitting parameter, we obtained a slightly better fit to the data, yielding T_{CW} $= -72(2)$ K and $\chi_0 = 0.54(3) \times 10^{-3}$ emu/mol.

Supplementary Note 3. ^{75}As NMR spectra and their analysis for Sr_2VO_3FeAs in a magnetic field $H \parallel (110)$

Supplementary Fig. 4 shows ^{75}As NMR spectra as a function of temperature for field orientations parallel to c (001) and the (110) directions, which were taken on a different single crystal of Sr_2VO_3FeAs. For $H \parallel c$, a sudden change in the Knight shift occurs at $T_0 \approx 155$ K, consistent with the results shown in Fig. 2 of the main text. For $H \parallel (110)$ we obtained the sudden shift of the NMR peak to high frequencies, similar to the results in $H \parallel a$. Neither peak splitting nor broadening were detected as shown in Supplementary Figs. 4c and 4d.

Supplementary Note 4. Comparison with the ^{75}As NMR behavior in other iron-based superconductors

In iron pnictides or chalcogenides, various types of the long-range spin and orbital orders are stabilized in parent compounds such as LaFeAsO and $BaFe_2As_2$. The most common parent phases are the C_2 orbital (nematic) order and the stripe-type C_2 antiferromagnetic (AFM) order [12]. The C_2 orbital order at T_S usually precedes the C_2 magnetic order at T_N ($T_S \geq T_N$), and depending on the coupling strength between these orders, their ordering temperatures differ in a wide range [13]. The most extreme case is FeSe where only a C_2 orbital ordering occurs, at $T_S = 90$ K, whereas the C_2 spin correlation fails to induce the static magnetic order [14]. There are some exceptions, however, exhibiting different types of AFM or orbital-ordered phases such as a bicollinear-AFM in FeTe [15], a double-Q C_4-AFM in (Ba,Na)Fe_2As_2 and (Ba,K)Fe_2As_2 [16–18], and a spin-reoriented-AFM orders in $Ba_{0.65}Na_{0.35}Fe_2As_2$ [19] as

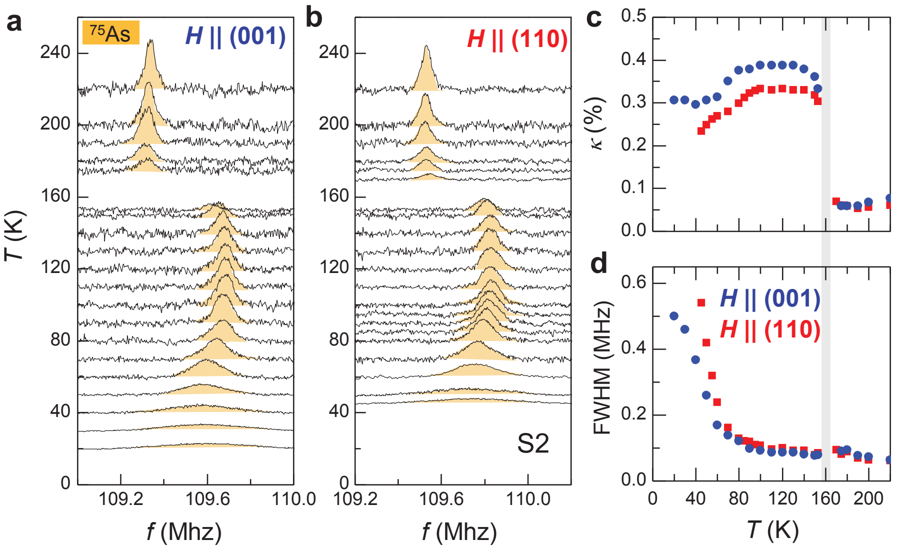

Supplementary Fig. 4: ^{75}As NMR spectra as a function of temperature, measured at $H = 15$ T at different field orientations **a** $H||(001)$ **b** $H||(110)$, respectively. Temperature dependence of **c** Knight shift and **c** linewidth for ^{75}As NMR spectra. The ^{75}As NMR spectra as a function of temperature for field orientations parallel to a (100) and the (110) direction show almost consistent behavior.

listed in the Supplementary Table 2.

This also includes the nonmagnetic collapsed C_4 phase in rare-earth doped $CaFe_2As_2$ as discussed in the supplementary section 1. These ground states have their own characteristic temperature dependences in the Knight shift and the spin-lattice relaxation rate $1/T_1T$, depending on the underlying spin or orbital structures, which can be categorized as shown in Supplementary Fig. 5. The observed NMR properties of Sr_2VO_3FeAs are highly distinct from those in other iron-pnictides or chalcogenides. Particularly, the spin pseudogap behavior, *i.e.*, dramatic suppression of $1/T_1T$ is a precursor of the C_4 symmetric order at T_0, as discussed in the main text, reflecting the unique role of the interfacial Fe-V interaction in the hetero-structured Sr_2VO_3FeAs.

Supplementary Table 2: Representative antiferromagnetic and orbital-ordered phases considered for iron pnictides and chalcogenides. The patterns of the orbital and spin orders, the corresponding transition temperatures (T_S) and T_N), and the behavior of NMR properties are listed in Supplementary Fig. 5.

Family	Compounds	Electronic ground state: Orbital		Electronic ground state: Spin		NMR results
11	FeSe	C_2	$T_S \sim 90$ K	-	-	**a** [14]
	FeTe [15]	C_2	$T_S \sim 67$ K	Bicollinear-AFM	-	-
111	NaFeAs	C_2	$T_S \sim 49$ K	C-type AFM	$T_N \sim 39$ K	**b** [20]
	LiFeAs	-	-	-	-	-
122	$BaFe_2As_2$	C_2	$T_S \sim 142$ K	C-type AFM	$T_N \sim 143$ K	**b** [21, 22]
	$SrFe_2As_2$	C_2	$T_S \sim 173$ K	C-type AFM	$T_N \sim 220$ K	**b** [23]
	$(Ca,Pr)Fe_2As_2$	Collapsed-C_4	$T_S \sim 65$ K	-	-	**c** [24, 25]
	$(Ba,Na)Fe_2As_2$ [26]	C_2	$T_{S1} \sim 80$ K	Double-Q AF in C_4-lattice	$T_{N1} \sim 80$ K	-
		C_4	$T_{S2} \sim 50$ K	Spin-reoriented C_4	$T_{N2} \sim 50$ K	-
1111	LaFeAsO	C_2	$T_S \sim 155$ K	C-type AFM	$T_N \sim 137$ K	**b** [27]
	LaFeAs(O,H)	C_2	$T_S \sim 95$ K	C-type AFM	$T_N \sim 89$ K	**b** [28]
21311	$Ca_2AlO_3Fe(As,P)$	-	-	AFM	$T_N \sim 70$ K	**b** [29]
	$Sr_2(Mg,Ti)O_3FeAs$	-	-	AFM	$T_N \sim 50$ K	**b** [30]
	Sr_2ScO_3FeAs [31]	-	-	AFM	$T_N \sim 35$ K	-
21311	Sr_2VO_3FeAs (our work)	C_4	$T_0 \sim 155$ K	Spin-gap-like	$T_{sg} \sim 220$ K	**d**
				C_4-AFM	$T_N \sim 45$ K	

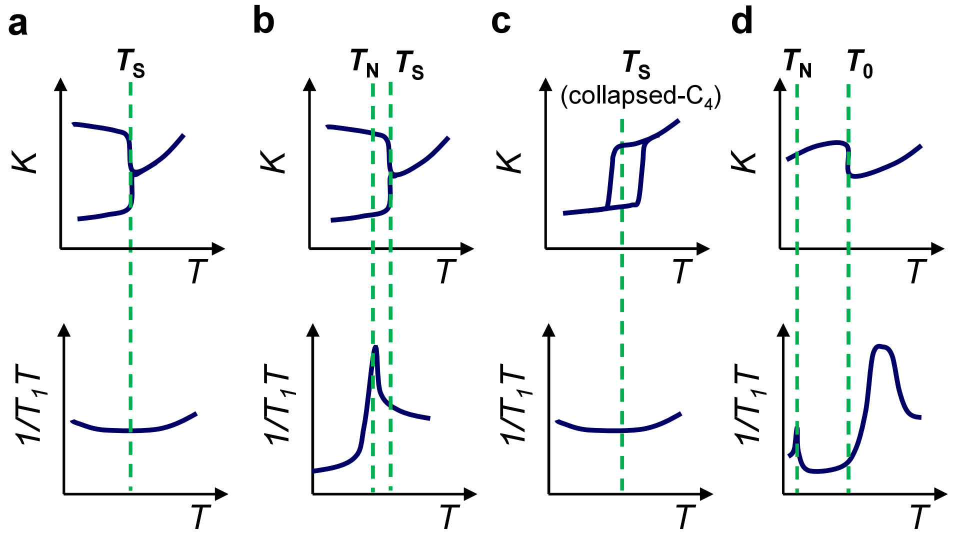

Supplementary Fig. 5: A cartoon illustration of various types of the temperature variation of the Knight shift(K) and the spin-lattice relaxation rate ($1/T1T$) in iron pnictides and chalcogenides. **a** The C_2 orbital ordered phase without any static AFM order in FeSe. **b** The C_2 orbital-ordered and AFM phases, the most commonly observed parent phase in AFe$_2$As$_2$, *Re*OFeAs, and A_2MO$_3$FeAs (A = alkaline earths, *Re* = rare earths, M = nonmagnetic metals). **c** The collapsed C_4 phase in (Ca,*Re*)Fe$_2$As$_2$. **d** Putative C_4 orbital-ordered and AFM phases in Sr$_2$VO$_3$FeAs (this work).

Supplementary Note 5. ^{75}As NMR peak splitting in ordered phases of iron pnictides

Symmetry analysis of the hyperfine coupling tensor. As described in Ref. [21], the hyperfine coupling between ^{75}As nucleus and Fe moments has different characters, depending on the symmetry breaking by the underlying spin/orbital orders. Since the As site is located above and below the center of Fe plaquette in the square lattice, the hyperfine field at the As site, H^{hf}, is determined by adding the contribution of the surrounding four Fe spins as expressed by

$$H_i^{hf} = \sum_{j\alpha\beta} B_{ij}^{\alpha\beta} s_j^{\alpha\beta}, \tag{1}$$

where i, j are Cartesian indices, x (a), y (b) or z (c), $\mathbf{s}^{\alpha\beta}$ is the spin at the plaquette site $\alpha\beta$, where $\alpha\beta$ takes four values $\pm1, \pm1$. $\hat{\mathbf{B}}^{\alpha\beta}$ is the hyperfine coupling tensor between the As nucleus and Fe moments at the $\alpha\beta$ site which can be given by symmetry rotations,

$$B_{ij}^{\alpha\beta} = e_i^{\alpha\beta} B_{ij}^0 e_j^{\alpha\beta}, \tag{2}$$

$$e^{++} = (1,1,1);\; e_i^{+-} = (1,-1,1);\; e_i^{-+} = (-1,1,1);\; e_i^{--} = (-1,-1,1). \tag{3}$$

Here, $\hat{\mathbf{B}}^0$ can be written using explicitly the underlying tetragonal symmetry,

$$\hat{\mathbf{B}}^0 = \begin{pmatrix} B_{aa} & B_{ab} & B_{ac} \\ B_{ba} & B_{bb} & B_{bc} \\ B_{ca} & B_{cb} & B_{cc} \end{pmatrix} = \begin{pmatrix} X & U & V \\ U & X & V \\ V & V & Z \end{pmatrix}. \tag{4}$$

For collinear structures, $\mathbf{s}^{\alpha\beta} = \sigma^{\alpha\beta}\mathbf{s}$, where $\mathbf{s} = (s_a,\, s_b,\, s_c)$. Then the formula can by simplified further as

$$H_i^{hf}(\mathbf{s}||j) = \sum_{\alpha\beta} e_i^{\alpha\beta} B_{ij}^0 e_j^{\alpha\beta} \sigma^{\alpha\beta}\mathbf{s} = B_{ij}^0 T_{ij}\mathbf{s}, \tag{5}$$

$$T_{ij} = \sum_{\alpha\beta} e_i^{\alpha\beta} e_j^{\alpha\beta} \sigma^{\alpha\beta} = \sum_{\alpha\beta} t_{ij}^{\alpha\beta} \sigma^{\alpha\beta}, \tag{6}$$

where the matrix T carries all information about a particular magnetic pattern. Here, the matrices t are

$$t^{++} = \begin{pmatrix} 1 & 1 & 1 \\ 1 & 1 & 1 \\ 1 & 1 & 1 \end{pmatrix};\; t^{+-} = \begin{pmatrix} 1 & -1 & 1 \\ -1 & 1 & -1 \\ 1 & -1 & 1 \end{pmatrix};\; t^{-+} = \begin{pmatrix} 1 & -1 & -1 \\ -1 & 1 & 1 \\ -1 & 1 & 1 \end{pmatrix};\; t^{--} = \begin{pmatrix} 1 & 1 & -1 \\ 1 & 1 & -1 \\ -1 & -1 & 1 \end{pmatrix}. \quad (7)$$

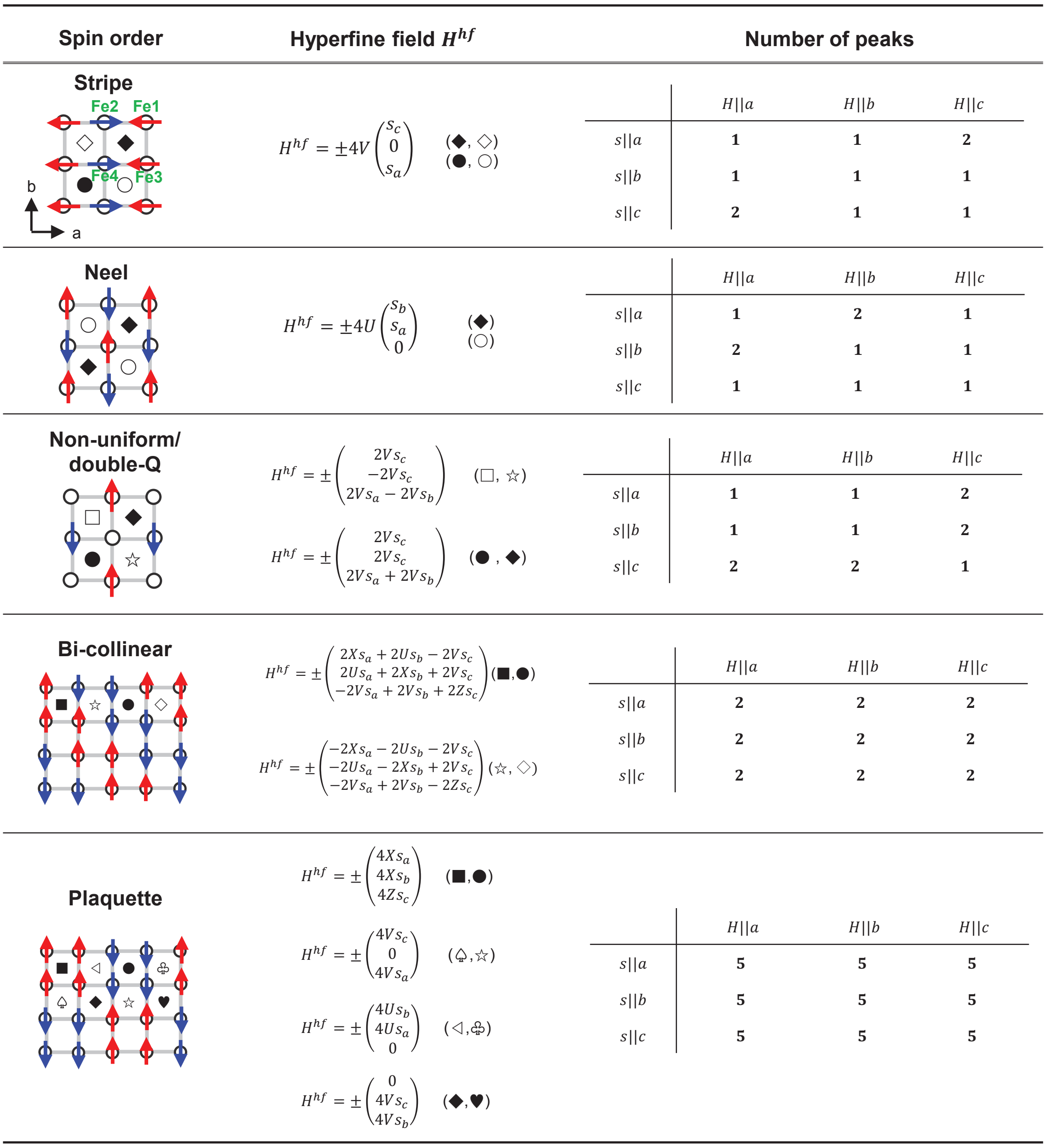

Supplementary Fig. 6: Schematic spin configurations of various antiferromagnetic (AFM) orders in iron pnictides and the corresponding hyperfine field (H_{hf}) at the As sites. Four neighboring Fe spins, s_i (i = 1,2,3,4), determining the H^{hf} at the As site in the center are indicated by arrows on top of the larger open circles as shown in the case of the stripe AFM. The same indexing scheme of s_i is used for calculating H^{hf} at the different As sites, indicated by smaller symbols, above (solid) and below (open) the Fe square lattice. The resulting NMR line splitting is also given for different orientations of spin s and external magnetic field H along the a, b, c axes.

NMR line splitting due to antiferromagnetic orders. In a magnetically ordered state, the Fe spins on an Fe_4 plaquette have a particular ordering, as illustrated in Supplementary Fig. 6. For example, in the stripe AFM state with the in-plane wave vector $\mathbf{Q} \approx (0, \pi)$, $\sigma^{\alpha\beta}$ can be expressed as $\begin{pmatrix} 1 & -1 \\ 1 & -1 \end{pmatrix}$. Accordingly, the characteristic matrix T and the corresponding hyperfine field H^{hf} are

$$T = \begin{pmatrix} 0 & 0 & 0 \\ 0 & 0 & 4 \\ 0 & 4 & 0 \end{pmatrix}; H^{hf} = \pm 4V \begin{pmatrix} s_c \\ 0 \\ s_a \end{pmatrix}. \tag{8}$$

Based on the above calculations, a stripe AFM with spin moments along the a-axis ($s \parallel a$) results in an internal hyperfine field along the c-axis. Thus the As nuclei, located above ($\bullet, \blacklozenge$ in Supplementary Fig. 6) and below ($\circ, \lozenge$ in Supplementary Fig. 6) the Fe plane, will experience the opposite internal hyperfine fields along the c-axis. When the external magnetic field H is applied along the c-axis ($H||c$), the total field at each As site is $H_{tot} = H \pm 4Vs$, which leads to two NMR peaks at different frequencies of $f = \gamma H_{tot} = \gamma(H \pm 4Vs)$. In contrast, with an external field along the a (or b) axis ($H \parallel a$ or $H \parallel b$), only one NMR peak is observed at a frequency $f = \gamma H_{tot} = \gamma\sqrt{H^2 + (4Vs)^2}$. Using the similar symmetry analysis, we can obtain the NMR frequency (f) induced by the stripe AFM orders with different orientations of the Fe spins ($s \parallel a$, $s \parallel b$, or $s \parallel c$) and external magnetic field orientations ($H \parallel a, H \parallel b, H \parallel c$), as summarized in the first panel of Supplementary Fig. 6. Following the same procedure, one can determine the net hyperfine coupling tensor ($B^0_{ij}T_{ij}$), and the net hyperfine field (H^{hf}) at the As sites for various types of the AFM phases with different spin configurations and also for different spin orientations, as listed in Supplementary Fig. 6.

For Neel-type correlations with $\mathbf{Q} \approx (\pi, \pi)$, the $\sigma^{\alpha\beta}$ is $\begin{pmatrix} + & - \\ - & + \end{pmatrix}$, and the corresponding matrix T and hyperfine field H^{hf} are

$$T = \begin{pmatrix} 0 & 4 & 0 \\ 4 & 0 & 0 \\ 0 & 0 & 0 \end{pmatrix}; H^{hf} = \pm 4U \begin{pmatrix} s_b \\ s_a \\ 0 \end{pmatrix}. \tag{9}$$

For the double-$\mathbf{Q}$ fluctuations with $\mathbf{Q} \approx (\pi, 0)$, $(0, \pi)$, two possible magnetic patterns are expected. Thus, the $\sigma^{\alpha\beta}$ for possible patterns are $\begin{pmatrix} 0 & + \\ - & 0 \end{pmatrix}$ and $\begin{pmatrix} + & 0 \\ 0 & - \end{pmatrix}$. The corresponding matrices T the hyperfine field H^{hf} at different sites are

$$T = \begin{pmatrix} 0 & 0 & 2 \\ 0 & 0 & -2 \\ 2 & -2 & 0 \end{pmatrix}, \begin{pmatrix} 0 & 0 & 2 \\ 0 & 0 & 2 \\ 2 & 2 & 0 \end{pmatrix}, \tag{10}$$

and

$$H^{hf} = \pm 2V \begin{pmatrix} s_c \\ -s_c \\ s_a - s_b \end{pmatrix}; \pm 2V \begin{pmatrix} s_c \\ s_c \\ s_a + s_b \end{pmatrix}. \tag{11}$$

For bi-collinear correlations, $\mathbf{Q} \approx (\pi, \pi)$, the possible patterns are $\begin{pmatrix} + & - \\ + & + \end{pmatrix}$ and $\begin{pmatrix} - & - \\ + & - \end{pmatrix}$. The corresponding matrices T the hyperfine field H^{hf} at different sites are

$$T = \begin{pmatrix} 2 & 2 & -2 \\ 2 & 2 & 2 \\ -2 & 2 & 2 \end{pmatrix}, \begin{pmatrix} -2 & -2 & -2 \\ -2 & -2 & 2 \\ -2 & 2 & -2 \end{pmatrix}, \tag{12}$$

and

$$H^{hf} = \pm 2 \begin{pmatrix} Xs_a + Us_b - Vs_c \\ Us_a + Xs_b + Vs_c \\ -Vs_a + Vs_b + Zs_c \end{pmatrix}; \pm 2 \begin{pmatrix} -Xs_a - Us_b - Vs_c \\ -Us_a - Xs_b + Vs_c \\ -Vs_a + Vs_b - Zs_c \end{pmatrix}. \tag{13}$$

For plaquette correlation, there are four patterns, $\begin{pmatrix} + & + \\ + & + \end{pmatrix}$, $\begin{pmatrix} + & + \\ - & - \end{pmatrix}$ and $\begin{pmatrix} + & - \\ + & - \end{pmatrix}$, and $\begin{pmatrix} + & - \\ - & + \end{pmatrix}$.
The corresponding matrices T the hyperfine field H^{hf} at different sites are

$$T = \begin{pmatrix} 4 & 0 & 0 \\ 0 & 4 & 0 \\ 0 & 0 & 4 \end{pmatrix}, \begin{pmatrix} 0 & 0 & 4 \\ 0 & 0 & 0 \\ 4 & 0 & 0 \end{pmatrix}, \begin{pmatrix} 0 & 4 & 0 \\ 4 & 0 & 0 \\ 0 & 0 & 0 \end{pmatrix}, \begin{pmatrix} 0 & 0 & 0 \\ 0 & 0 & 4 \\ 0 & 4 & 0 \end{pmatrix}, \tag{14}$$

and

$$H^{hf} = \pm 4 \begin{pmatrix} Xs_a \\ Xs_b \\ Zs_c \end{pmatrix}; \pm 4V \begin{pmatrix} s_c \\ 0 \\ s_a \end{pmatrix}; \pm 4U \begin{pmatrix} s_b \\ s_a \\ 0 \end{pmatrix}; \pm 4V \begin{pmatrix} 0 \\ s_c \\ s_b \end{pmatrix}. \tag{15}$$

Now we discuss the possible 75^As NMR line splitting for various types of AFM orders, which have been identified experimentally or suggested theoretically in iron pnictides, including the stripe-AFM, the bicollinear-AFM, the double-Q C_4-AFM, the spin-reoriented-AFM, the non-collinear AFM, and the plaquette AFM orders. As summarized in Supplementary Fig. 6, in some cases with a small magnetic unit cell, such as stripe or Neel orders, the hyperfine field H^{hf} points along either the a or c axes, which results in NMR line splitting for either $H \parallel c$ or $H \parallel a$. In some cases, such as bicollinear and plaquette AFM orders, there are more than two inequivalent As sites having different hyperfine coupling tensors and thus different net H^{hf} fields along the a or c axes. Then this results in an NMR line splitting under both $H \parallel c$ and $H \parallel a$, which is not consistent with the experimental results for Sr_2VO_3FeAs. There are only two cases where no hyperfine field is generated on the As site, namely, the stripe AFM phase with the spins aligned in-plane and normal to the AFM ordering vector ($\mathbf{s} \perp \mathbf{Q} = (1, 0)$) and the Neel AFM phase with the out-of-plane spins ($\mathbf{s}||c$). In this case no splitting of the As NMR line is expected. However even in this case (as in any other magnetically-ordered state) we should have observed considerable peak broadening of ^{75}As NMR spectra due to the directional fluctuation of Fe spins in the AFM phase or a diverging behavior of $1/T_1T$ across T_0, which were not observed in the experiments. Therefore, we can conclude that no long-range magnetic order emerges at T_0.

NMR peak splitting due to nematic orders. Even without a long-range magnetic order, the NMR line splitting can be induced when the local symmetry at the As site is broken in the nematic phases. As described in the case of FeSe [14], the Knight shifts $K_{\alpha\alpha} = A^{hf}_{\alpha\alpha}\chi_{\alpha\alpha}$ ($\alpha = x, y, z$) is affected by the nematic order, which breaks the in-plane rotational symmetry and introduces two non-equivalent directions in the plane. In this case, as the spin fluctuation or orbital occupation becomes anisotropic in the plane, indicated by the d_{xz} or d_{yz} orbital in Supplementary Fig. 7, the spin susceptibility (χ) or the hyperfine coupling (A^{hf}) at the As site also become anisotropic, *i.e.*, $\chi_{xx} \neq \chi_{yy}$ and/or $A^{hf}_{xx} \neq A^{hf}_{yy}$. In this case, the isotropic (K_{av}) and anisotropic (ΔK) parts of the in-plane Knight shift are given as

$$K_{av} = 1/2(A^{hf}_{xx} + A^{hf}_{yy})(\chi_{xx} + \chi_{yy}) + 1/2(A^{hf}_{xx} - A^{hf}_{yy})(\chi_{xx} - \chi_{yy}), \tag{16}$$

and

$$\Delta K = 1/2(A^{hf}_{xx} + A^{hf}_{yy})(\chi_{xx} - \chi_{yy}) + 1/2(A^{hf}_{xx} - A^{hf}_{yy})(\chi_{xx} + \chi_{yy}). \tag{17}$$

For example, in the conventional stripe nematic order, the x and y directions are aligned along one of the crystallographic directions, *e.g*, $x \parallel a$ and $y \parallel b$. In this detwinned case, there is one equivalent As site, and thus As NMR line will be shifted differently for $H \parallel a$ and $H \parallel b$. In reality, when external magnetic field is applied *e.g.* $H \parallel a$, crystals are twined with two domains of $H \parallel a$ and $H \parallel b$ as the stripe nematic order develops, which results in the As NMR line splitting. For $H \parallel (110)$, on the other hand, the isotropic part, dominated by the average susceptibility, $1/2(\chi_{xx} + \chi_{yy})$, and the average hyperfine coupling constant, $1/2(A^{hf}_{xx} + A^{hf}_{yy})$, is measured, which turns out to be almost the same across the stripe nematic transition, as experimentally confirmed in FeSe and also in LaFeAsO. This behavior is not consistent with what observed in Sr_2VO_3FeAs as shown in Fig. 2 of the main text.

We can consider nematic or vestigial phases corresponding to other types of AFM spin orders, in the same spirit as the standard C_2 nematicity, breaking the C_4 tetragonal symmetry but preserving the $O(3)$ spin-rotational symmetry, is a "vestige" of the stripe AFM phase. For examples, one can consider vestigial phases corresponding to the more complex AFM phases, such as bicollinear or plaquette AFM orders. For the bicollinear nematic phase, the C_4 tetragonal symmetry is broken by the next-nearest neighbor bond order along the diagonal direction of the Fe square lattice, indicated by the blue oval in Supplementary Fig. 7. This order must trigger an orbital order with different

occupations between d_{xz+yz} and d_{xz-yz} states. By further developing the nearest neighbor bond order, indicated by the red oval in Supplementary Fig. 7, the reflection and the translation symmetries are broken. In this case, the line splitting is expected for twinned crystals, similar to the stripe nematic case, but not for $H \parallel (100)$ but for $H \parallel (110)$ as summarized in Supplementary Fig. 7. The vestigial phase for the plaquette AFM order has more than one inequivalent As sites, and must trigger an As NMR peak splitting in any magnetic field orientation. Note that in both cases the generated orbital imbalance between the corresponding orbital combinations of Fe $d_{xz} \pm d_{yz}$ is expected to be of the same order of magnitude as in the stripe-nematic case, so also a the peak splitting for $H \parallel (110)$ should be detectable. This is however not observed in experiments.

As a possible candidate we consider the vestigial phase for the double-Q AFM phase, which generate a checkerboard-type combination of two charge density waves. In this case, the charge density for C_4 orbital states such as d_{xy} state is unequal between odd and even sites of the Fe square lattice as shown in Supplementary Fig. 7. Thus the time-reversal and C_4 tetragonal symmetries are preserved, but the translational symmetry is broken by unit-cell doubling in the Fe-only lattice. Note however that this unit-cell doubling exactly matches the unit-cell doubling due the As sites above and below the Fe square lattice. Thus the vestigial phase for the nonuniform double-Q phase has a C_4 symmetric and intra-unit-cell order in the FeAs layers.

In this case, each As then has two nonmagnetic Fe neighbors, and two paramagnetic, with non-zero local moments

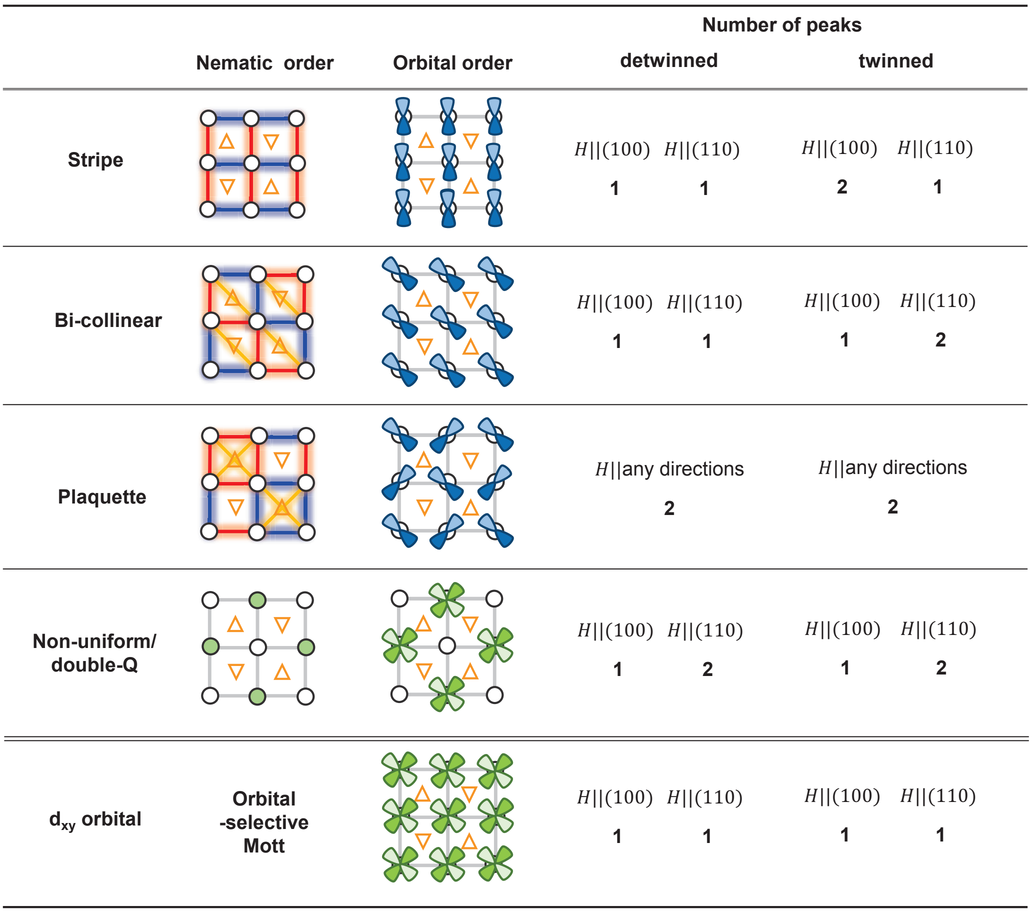

Supplementary Fig. 7: Schematic illustration of various nematic orders and the possible accompanying orbital orders in iron pnictides. The nearest (next-nearest) neighbor ferromagnetic bonds are indicated by thick red (blue) ovals. For the double-Q nematic order, the magnetically active sites are indicated by filled (orange) circles; their charge and/or orbital state can differ from that of the nonmagentic (open circles) states. For the orbital selective Mott-state, the orbital population of each state is the same but different from that in the high-temperature phase. We illustrated orbital ordering by drawing an orbital whose population could be different above and below the transition. The expected number of the As NMR frequencies for detwinned/twinned crystal are listed for each relevant external field orientation.

that are fluctuating so as to preserve short-range spin correlations among themselves. This affects the Fe-As hybridization and modifies the ^{75}As Knight shift as observed in our experiments (Fig. 2 and Supplementary Fig. 4). Strictly speaking, the ^{75}As line splitting could occur for a field parallel to (110), since half of As in this phase have paramagnetic neighbors along (110), and half along $(1\bar{1}0)$. However, we observed the ^{75}As line shift nearly independent on the filed directions, indicating that it results from the change of the isotropic Fermi-contact and core-polarization terms, and not dipole or orbital ones. Therefore it is likely that the signal is dominated by the former interaction, in which case the expected splitting can be vanishingly small, below the experimental resolution.

Indirect support to this scenario comes from LDA+DMFT calculations, which can address the difference between the paramagnetic and nonmagnetic Fe, since they account for local spin fluctuation (albeit not short-range correlations). In typical FeSCs, the relative d-orbital occupation changes slightly ($\sim$ 3-5%) [32], although the average Fe-As hybridization changes. This is also the case of Sr_2VO_3FeAs, in which the d-orbital occupations, obtained by DFT+DMFT calculations, differ by only 4-8% with respect to the DFT results. This is much smaller than the typical difference ($\sim$ 20%) in orbital occupations between d_{xz} and d_{yz} states for the C_2 nematic phases [33]. This observation again supports the idea that the shift of the NMR lines in the vestigial phase may be much larger than it splitting. An advantage of this scenario is that it is driven by the same spin fluctuations at $Q = (0, \pi)$ and $(\pi, 0)$ that are present above T_0 and manifest themselves *via* critically enhanced relaxation rate $1/T_1T$ above $\sim$ 200 K.

One can envision a fully nonmagnetic phase transition changing orbital occupations at $T_0 = 155$ K, such as an orbital-selective Mott-transition with delocalized d_{xy} bands above T_0 into gapped Hubbard states below. In this case, the orbital occupation of, *e.g.*, d_{xy} states is changes for all the Fe sites, keeping the C_4 symmetry intact, as shown in Supplementary Fig. 7. This would result in a nearly isotropic As NMR peak shift without splitting or broadening, consistent with the experiments. However, it seems somewhat counterintuitive that the critical stripe-like fluctuations observed above the transition disappear without trace at T_0; so far materials that were proposed as candidates for such an orbital-selective Mott transition have been either much more strongly correlated than low-doping pnictides (such as isostructural chalcogenides) or severely underdoped and thus closer to half-filling (KFe_2As_2). At the current stage, it is not clear which is the true order below T_0, the vestigial non-uniform double-Q phase or an orbital-selective Mott transition of C_4 orbitals. Nevertheless, the observed C_4 symmetric and intra-unit-cell order in Sr_2VO_3FeAs is unprecedented in iron pnictides, emphasizing the importance of the interfacial Fe-V interactions in the heterostructure.

Supplementary Note 6. Spin-lattice relaxation rate in the normal state

The spin-lattice relaxation rate can be derived based on the fluctuation-dissipation theorem. The $1/T_1$ is usually described in terms of the hyperfine field fluctuations perpendicular to the external magnetic fields [21, 34, 35].

$$\left(\frac{1}{T_1}\right)_z = \frac{(\mu_0\gamma_N)^2}{2}\int_{\infty}^{-\infty} dt(< H_{hf,x}(t), H_{hf,x}(0) > + < H_{hf,y}(t), H_{hf,y}(0) >)e^{i\omega_0 t}$$

$$= (\mu_0\gamma_N)^2(|H_{hf,x}(\omega_0)|^2 + |H_{hf,y}(\omega_0)|^2) \tag{18}$$

Each As has four nearest neighbor Fe immediately above or below. Since the magnetic anisotropy in FeSC is at least one order of magnitude weaker that the isotropic coupling, at high temperatures the four Fe spins around an As will fluctuate coherently, respecting a particular pattern, and the the hyperfine field on As will fluctuate correspondingly. For an external field direction $\hat{\mathbf{n}}$ the relaxation rate will be defined by

$$\left(\frac{1}{T_1}\right)_{\hat{\mathbf{n}}} = (\mu_0\gamma_N)^2\left\langle|\mathbf{H}_{hf}(\omega_0)\times\hat{\mathbf{n}}|^2\right\rangle \tag{19}$$

Where $\langle\rangle$ is averaging over all possible types of fluctuations. For instance, for the stripe fluctuations we average over two types of spin plaquettes, $\begin{pmatrix} + & - \\ + & - \end{pmatrix}$ and $\begin{pmatrix} + & + \\ - & - \end{pmatrix}$, and over all spin directions (collinear in the plaquette). Let us first do the some rearranging:

$$\left\langle|\mathbf{H}^{hf}\times\hat{\mathbf{n}}|^2\right\rangle = \sum_{ij}\left\langle(\delta_{ij} - n_i n_j)H_i^{hf}H_j^{hf}\right\rangle_{dir} = \sum_{ij}(\delta_{ij} - n_i n_j)\left\langle H_i^{hf}H_j^{hf}\right\rangle_{dir}, \tag{20}$$

where

$$\left\langle H_i^{hf}H_j^{hf}\right\rangle = \sum_{ijkl\alpha\beta\gamma\delta} B_{ik}^{\alpha\beta}s_k^{\alpha\beta}B_{jl}^{\gamma\delta}s_l^{\gamma\delta} = \sum_k B_{ik}^0 T_{ik}B_{jk}^0 T_{jk}. \tag{21}$$

For AFM stripe spin fluctuations with $\mathbf{Q} \approx (0, \pi)$, $\sigma^{\alpha\beta}$ takes two values, $\begin{pmatrix} + & - \\ + & - \end{pmatrix}$ and $\begin{pmatrix} + & + \\ - & - \end{pmatrix}$. The corresponding matrices T are

$$T = \begin{pmatrix} 0 & 0 & 0 \\ 0 & 0 & 4 \\ 0 & 4 & 0 \end{pmatrix}, \begin{pmatrix} 0 & 0 & 4 \\ 0 & 0 & 0 \\ 4 & 0 & 0 \end{pmatrix}. \tag{22}$$

This immediately gives us

$$\left\langle H_i^{hf} H_j^{hf} \right\rangle = \frac{1}{2} \left[\begin{pmatrix} 16V^2 & 0 & 0 \\ 0 & 0 & 0 \\ 0 & 0 & 16V^2 \end{pmatrix} + \begin{pmatrix} 0 & 0 & 0 \\ 0 & 16V^2 & 0 \\ 0 & 0 & 16V^2 \end{pmatrix} \right] = \begin{pmatrix} 8V^2 & 0 & 0 \\ 0 & 8V^2 & 0 \\ 0 & 0 & 16V^2 \end{pmatrix}. \tag{23}$$

For the in- and out of plane relaxation rate, $(1/T_1)_{H||a} = (1/T_1)_{H||b} \propto Tr \left\langle H_i^{hf} H_j^{hf} \right\rangle - \left\langle H_1^{hf} H_1^{hf} \right\rangle = 24V^2$, $(1/T_1)_{H||c} \propto Tr \left\langle H_i^{hf} H_j^{hf} \right\rangle - \left\langle H_3^{hf} H_3^{hf} \right\rangle = 16V^2$, the anisotropy ratio is thus 1.5, as has been experimentally observed in many FeSCs.

For Neel-type correlations, $\mathbf{Q} \approx (\pi, \pi)$, the only possible pattern is $\begin{pmatrix} + & - \\ - & + \end{pmatrix}$,

$$T = \begin{pmatrix} 0 & 4 & 0 \\ 4 & 0 & 0 \\ 0 & 0 & 0 \end{pmatrix}, \tag{24}$$

$$\left\langle H_i^{hf} H_j^{hf} \right\rangle = \begin{pmatrix} 16V^2 & 0 & 0 \\ 0 & 16V^2 & 0 \\ 0 & 0 & 0 \end{pmatrix}, \tag{25}$$

and $(1/T_1)_{H||a} = (1/T_1)_{H||b} \propto Tr \left\langle H_i^{hf} H_j^{hf} \right\rangle - \left\langle H_1^{hf} H_1^{hf} \right\rangle = 16V^2$, $(1/T_1)_{H||c} \propto Tr \left\langle H_i^{hf} H_j^{hf} \right\rangle - \left\langle H_3^{hf} H_3^{hf} \right\rangle = 32V^2$, the anisotropy ratio is 0.5.

For the double-$\mathbf{Q}$ fluctuations both wave vectors are excited equally, $\mathbf{Q} \approx (\pi, 0)$, $(0, \pi)$, the possible patterns are $\begin{pmatrix} 0 & - \\ + & 0 \end{pmatrix}$ and $\begin{pmatrix} + & 0 \\ 0 & - \end{pmatrix}$,

$$T = \begin{pmatrix} 0 & 0 & -2 \\ 0 & 0 & 2 \\ -2 & 2 & 0 \end{pmatrix}, \begin{pmatrix} 0 & 0 & 2 \\ 0 & 0 & 2 \\ 2 & 2 & 0 \end{pmatrix}, \tag{26}$$

$$\left\langle H_i^{hf} H_j^{hf} \right\rangle = \frac{1}{2} \left[\begin{pmatrix} 4V^2 & 4V^2 & 0 \\ 4V^2 & 4V^2 & 0 \\ 0 & 0 & 8V^2 \end{pmatrix} + \begin{pmatrix} 4V^2 & -4V^2 & 0 \\ -4V^2 & 4V^2 & 0 \\ 0 & 0 & 8V^2 \end{pmatrix} \right] = \begin{pmatrix} 4V^2 & 0 & 0 \\ 0 & 4V^2 & 0 \\ 0 & 0 & 8V^2 \end{pmatrix}. \tag{27}$$

$(1/T_1)_{H||a} = (1/T_1)_{H||b} \propto Tr \left\langle H_i^{hf} H_j^{hf} \right\rangle - \left\langle H_1^{hf} H_1^{hf} \right\rangle = 12V^2$, $(1/T_1)_{H||c} \propto Tr \left\langle H_i^{hf} H_j^{hf} \right\rangle - \left\langle H_3^{hf} H_3^{hf} \right\rangle = 8V^2$, the anisotropy ratio is again 1.5, as expected from the fact that the fluctuations have the same wave vector as for the stripe case

Finally, for bi-collinear correlations, $\mathbf{Q} \approx (\pi, \pi)$, the possible patterns are $\begin{pmatrix} + & - \\ + & + \end{pmatrix}$ and three partners obtained by rotating the "$-$" site. Now

$$T = \begin{pmatrix} 2 & 2 & -2 \\ 2 & 2 & 2 \\ -2 & 2 & 2 \end{pmatrix}, \begin{pmatrix} 2 & 2 & 2 \\ 2 & 2 & -2 \\ 2 & -2 & 2 \end{pmatrix}, \begin{pmatrix} 2 & -2 & 2 \\ -2 & 2 & 2 \\ 2 & 2 & 2 \end{pmatrix}, \begin{pmatrix} 2 & -2 & -2 \\ -2 & 2 & -2 \\ -2 & -2 & 2 \end{pmatrix}, \tag{28}$$

$$\left\langle H_i^{hf} H_j^{hf} \right\rangle = \begin{pmatrix} 4X^2 + o & 0 & 0 \\ 0 & 4X^2 + o & 0 \\ 0 & 0 & 4Z^2 + o \end{pmatrix}. \tag{29}$$

In the last line, o means combinations of the off-diagonal couplings, which are much smaller than the diagonal one. The full calculation renders $(1/T_1)_{H||a} = (1/T_1)_{H||b} \propto 4X^2+4Z^2+4U^2+12V^2$, $(1/T_1)_{H||c} \propto 8X^2+8U^2+8V^2$, the anisotropy thus is $(X^2+Z^2+U^2+3V^2)/2(X^2+U^2+V^2) \approx (X^2+Z^2)/2X^2$, and thus depends on the anisotropy of the diagonal hyperfine coupling, which, if the Fermi-contact coupling dominates, is small.

For plaquette correlation, there are four patterns $\begin{pmatrix} + & + \\ + & + \end{pmatrix}$, $\begin{pmatrix} + & + \\ - & - \end{pmatrix}$, $\begin{pmatrix} + & - \\ + & - \end{pmatrix}$, and $\begin{pmatrix} + & - \\ - & + \end{pmatrix}$, which have the same probability. Correspondingly, we have

$$T = \begin{pmatrix} 4 & 0 & 0 \\ 0 & 4 & 0 \\ 0 & 0 & 4 \end{pmatrix}, \begin{pmatrix} 0 & 0 & 4 \\ 0 & 0 & 0 \\ 4 & 0 & 0 \end{pmatrix}, \begin{pmatrix} 0 & 0 & 0 \\ 0 & 0 & 4 \\ 0 & 4 & 0 \end{pmatrix}, \begin{pmatrix} 0 & 4 & 0 \\ 4 & 0 & 0 \\ 0 & 0 & 0 \end{pmatrix}, \tag{30}$$

$$\left\langle H_i^{hf} H_j^{hf} \right\rangle = \frac{1}{4} \left[\begin{pmatrix} 16X^2 & 0 & 0 \\ 0 & 16X^2 & 0 \\ 0 & 0 & 16Z^2 \end{pmatrix} + \begin{pmatrix} 16V^2 & 0 & 0 \\ 0 & 0 & 0 \\ 0 & 0 & 16V^2 \end{pmatrix} + \begin{pmatrix} 0 & 0 & 0 \\ 0 & 16V^2 & 0 \\ 0 & 0 & 16V^2 \end{pmatrix} + \begin{pmatrix} 16U^2 & 0 & 0 \\ 0 & 16U^2 & 0 \\ 0 & 0 & 0 \end{pmatrix} \right] \tag{31}$$

$$= \begin{pmatrix} 4X^2+o & 0 & 0 \\ 0 & 4X^2+o & 0 \\ 0 & 0 & 4Z^2+o \end{pmatrix}. \tag{32}$$

The result reads: $(1/T_1)_{H||a} = (1/T_1)_{H||b} \propto 8X^2+8Z^2+12V^2$, $\propto 4X^2+4Z^2+4U^2+12V^2$ $(1/T_1)_{H||c} \propto 16X^2+8V^2$, $\propto 8X^2+8U^2+8V^2$ the anisotropy thus is $(2X^2+2Z^2+3V^2)/2(2X^2+V^2) \approx (X^2+Z^2)/2X^2$,$(4X^2+4Z^2+4U^2+12V^2)/(8X^2+8U^2+8V^2) \approx (X^2+Z^2)/2X^2$, same as for the bilinear fluctuations.

Supplementary Fig. 8 shows the temperature dependence of the anisotropy ratio $R = T_{1,a}^{-1}/T_{1,c}^{-1}$ in our measurements. The anisotropy at high temperatures is $R \equiv T_{1,a}^{-1}/T_{1,c}^{-1} \approx 1.5$, similar to that found in other iron pnictides [34, 36]. This observation strongly suggest that the $(\pi, 0)$ correlations of isotopic Fe spins are dominating in Sr_2VO_3FeAs at the high temperatures, as in the case of other iron pnictides. However, with lowering temperature, R is strongly enhanced up to $R \sim 6$ near T_0 (presumably, reflecting magnetocrystalline anisotropy that makes the incipient critical temperature be different for different spin directions) before rapidly dropping to $R \sim 1$ below T_0. R remains almost the same with further lowering temperatures, which never recover back to $R \sim 1.5$ near $T_N \approx 45$ K. This result implies that the dominant spin fluctuations in that temperature range are not of stripe-type and herald an onset of

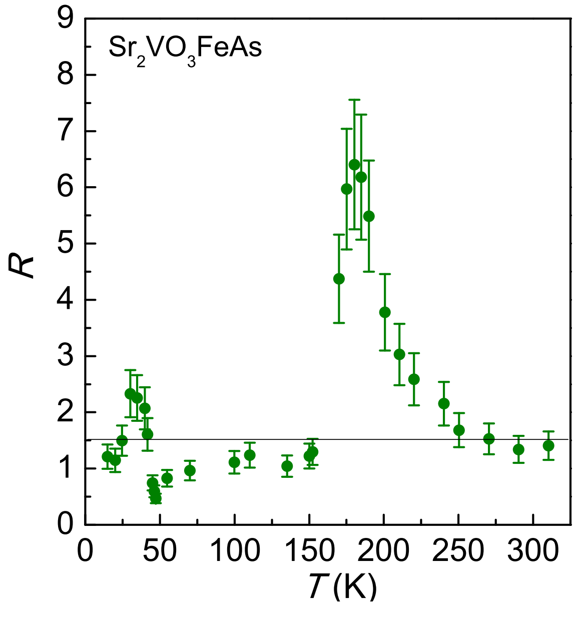

Supplementary Fig. 8: The anisotropy of spin-lattice relaxation rate $R \equiv T_{1,a}^{-1}/T_{1,c}^{-1}$ as a function of temperature. The horizontal line corresponds to the expected value for the stripe AFM correlation.

a spin order with a Q different from $(0, \pi)$. The long-pitch spin density wave with $Q = (1/8, 1/8, 0)$, suggested in the recent neutron powder diffraction [3], is a viable candidate. These results clearly demonstrate that not only the unusual C_4 symmetric transition at T_0, but also the magnetic transition at T_N is highly distinct from those found in other iron pnictides.

Supplementary Note 7. The ordered Fe magnetic moment below T_N = 45 K

From the full width at half maximum (FWHM) the As NMR peak $\sim$ 0.5 MHz , as shown in Fig. 2c in the main text, one can roughly estimate the ordered moment of Fe at low temperatures. For the spin density wave phase with $\mathbf{Q}$ = (1/8, 1/8, 0), the As environment is spin-imbalanced, and thus the diagonal hyperfine coupling will be dominant. In order to estimate the diagonal Fe-As hyperfine coupling, we have performed WIEN2k calculations keeping the V sublattice in the Mott insulating (antiferromagnetic) state and all Fe equivalent and ferromagnetic. We have performed both GGA and LDA calculations, which allow us to sample more magnetic moments, and also scaled the exchange field with different coefficients as described in Ref. [37]. The calculated hyperfine filed was then rescaled back with the inverse coefficient. In Supplementary Fig. 9 we plotted the average values of hyperfine field at the two inequivalent As sites, which experience induced by V hyperfine fields of the opposite signs. We note that the coupling comes mostly from the Fermi-contact term, the core polarization was taken into account but was smaller.

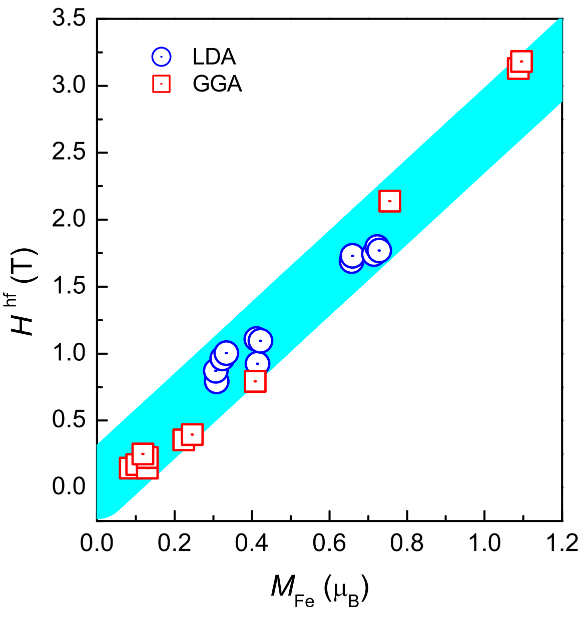

Supplementary Fig. 9: Calculated induced contact hyperfine field on the As site as a function of the net Fe magnetic moment.

The resulting diagonal coupling is $4A_{hf} \approx 2.5$ T/μ_B, which is somewhat larger than the typical off-diagonal hyperfine coupling $4A_{hf} \approx 1.7$ T/μ_B [21]. For a spiral with $\mathbf{Q}$ = (1/8, 1/8, 0) we would expect a net polarization around As to be $\sim$ 0.6 M, where M is the Fe moment. Using the nuclear gyromagnetic ratio 7.292 MHz/T for As and $4A_{hf} \approx$2.5 T/μ_B, the internal field H_{in} of As sites can be estimated to be $H_{in} = 4A_{hf} \times 0.6 M_0 \sim$ 800 Oe. This corresponds to the upper bound of Fe moments $\sim$ 0.05 μ_B, an order of magnitude smaller than found in other iron pnictides. Such a small Fe moments are also consistent with the estimates from neutron powder diffraction [3] and μSR spectroscopy [31].

In the previous Fe Mössbauer spectroscopy studies on polycrystalline Sr_2VO_3FeAs samples [1, 31], negligible line splitting or broadening was observed, which has been taken as experimental evidence of no static Fe spin ordering. However, the Fe Mössbauer spectra of polycrystalline Sr_2VO_3FeAs samples have a double peak structure and also a large FWHM already at room temperature, which hampers observation of magnetic ordering with such a small Fe moment less than 0.05 μ_B. As shown in the case of the isostructural compound Sr_2ScO_3FeAs, the line broadening of the Fe Mössbauer spectra is not at all obvious when the ordered moments is as small as $\sim$ 0.05 μ_B [31]. In strong contrast, the ^{75}As NMR line broadening observed at high temperature is an order of magnitude smaller than found

below T_N, which allow us to detect the Fe magnetic ordering of Sr_2VO_3FeAs single crystal in our study. From this comparison, one can say that, although it is not direct probe, ^{75}As NMR is much more sensitive to the ordering of small Fe moments in Sr_2VO_3FeAs, than Fe Mössbauer spectroscopy.

[1] G. H. Cao *et al.*, "Self-doping effect and successive magnetic transitions in superconducting $Sr_2VFeAsO_3$," Phys. Rev. B **82**, 104518 (2010).

[2] S. Tatematsu *et al.*, "Magnetic ordering in V-layers of the superconducting system of $Sr_2VFeAsO_3$," J. Phys. Soc. Jpn. **79**, 123712 (2010).

[3] M. Tegel *et al.*, "Possible magnetic order and suppression of superconductivity by V doping in Sr_2VO_3FeAs," Phys. Rev. B **82**, 140507 (2010).

[4] S. Kasahara *et al.*, "Electronic nematicity above the structural and superconducting transition in $BaFe_2(As_{1-x}P_x)_2$," Nature **486**, 382 (2012).

[5] M. A. Tanatar *et al.*, "Direct imaging of the structural domains in the iron pnictides AFe_2As_2 (A=Ca,Sr,Ba)," Phys. Rev. B **79**, 180508 (2009).

[6] M. Rotter *et al.*, "Spin-density-wave anomaly at 140 K in the ternary iron arsenide $BaFe_2As_2$," Phys. Rev. B **78**, 020503 (2008).

[7] N. Qureshi *et al.*, "Crystal and magnetic structure of the oxypnictide superconductor $LaFeAsO_{1-x}F_x$: A neutron-diffraction study," Phys. Rev. B **82**, 184521 (2010).

[8] R. Klingeler *et al.*, "Local antiferromagnetic correlations in the iron pnictide superconductors $LaFeAsO_{1-x}F_x$ and $Ca(Fe_{1-x}Co_x)_2As_2$ as seen via normal-state susceptibility," Phys. Rev. B **81**, 024506 (2010).

[9] G. F. Chen *et al.*, "Multiple phase transitions in single-crystalline $Na_{1-\delta}FeAs$," Phys. Rev. Lett. **102**, 227004 (2009).

[10] D. C. Johnston, "The puzzle of high temperature superconductivity in layered iron pnictides and chalcogenides," Advances in Physics **59**, 803 (2010).

[11] L. Y. Xing *et al.*, "Observation of non-Fermi liquid behavior in hole-doped $LiFe_{1-x}V_xAs$," Phys. Rev. B **94**, 094524 (2016).

[12] M. D. Lumsden and A. D. Christianson *et al.* "Magnetism in Fe-based superconductors," J. Phys. Condens. Matter **22**, 203203 (2010).

[13] R. M. Fernandes *et al.*, "What drives nematic order in iron-based superconductors?," Nat. Phys. **10**, 97-104 (2014).

[14] S.-H. Baek *et al.*, "Orbital-driven nematicity in FeSe," Nat. Mater. **14**, 210 (2015).

[15] F. Ma *et al.*, "First-principles calculations of the electronic structure of tetragonal α-FeTe and α-FeSe crystals: evidence for a bicollinear antiferromagnetic order," Phys. Rev. Lett. **102**, 177003 (2009).

[16] S. Avci *et al.*, "Magnetically driven suppression of nematic order in an iron-based superconductor," Nat. Commun. **5**, 3845 (2014).

[17] A. E. Böhmer *et al.*, "Superconductivity-induced re-entrance of the orthorhombic distortion in $Ba_{1-x}K_xFe_2As_2$," Nat. Commun. **6**, 7911 (2015).

[18] J. M. Allred *et al.*, "Double-Q spin-density wave in iron arsenide superconductors," Nat. Phys. **12**, 493 (2016).

[19] F. Waβer *et al.*, "Spin reorientation in $Ba_{0.65}Na_{0.35}Fe_2As_2$ studied by single-crystal neutron diffraction," Phys. Rev. B **91**, 060505 (2015).

[20] K. Kitagawa *et al.*, "Crossover from commensurate to incommensurate antiferromagnetism in stoichiometric NaFeAs revealed by single-crystal ^{23}Na,^{75}As-NMR experiments," J. Phys. Soc. Jpn. **80**, 033705 (2011).

[21] K. Kitagawa *et al.*, "Commensurate itinerant antiferromagnetism in $BaFe_2As_2$: ^{75}As-NMR studies on a self-flux grown single crystal," J. Phys. Soc. Jpn. **77**, 114709 (2008).

[22] F. L. Ning *et al.*, "Contrasting spin dynamics between underdoped and overdoped $Ba(Fe_{1-x}Co_x)_2As_2$," Phys. Rev. Lett. **104**, 037001 (2010).

[23] K. Kitagawa *et al.* "Antiferromagnetism of $SrFe_2As_2$ studied by single-crystal ^{75}As-NMR," J. Phys. Soc. Jpn. **78**, 063706 (2009).

[24] M. Long *et al.* "Quenched Fe moment in the collapsed tetragonal phase of $Ca_{1-x}Pr_xFe_2As_2$," Chin. Phys. B **22**, 057401 (2013).

[25] Y. Furukawa *et al.*, "Suppression of electron correlations in the collapsed tetragonal phase of $CaFe_2As_2$ under ambient pressure demonstrated by ^{75}As NMR/NQR measurements," Phys. Rev. B **89**, 121109 (2014).

[26] S. Avci *et al.*, "Magnetically driven suppression of nematic order in an iron-based superconductor," Nat. Commun. **10**, 4845 (2013).

[27] Y. Nakai *et al.*, "Evolution from itinerant antiferromagnet to unconventional superconductor with fluorine doping in $LaFeAs(O_{1-x}F_x)$ revealed by ^{75}As and ^{139}La nuclear magnetic resonance," J. Phys. Soc. Jpn. **77**, 073701 (2008).

[28] R. Sakurai *et al.*, "Quantum critical behavior in heavily doped $LaFeAsO_{1-x}H_x$ pnictide superconductors analyzed using nuclear magnetic resonance," Phys. Rev. B **91**, 064509 (2015).

[29] H. Kinouchi *et al.*, "Antiferromagnetic spin fluctuations and unconventional nodeless superconductivity in an iron-based new superconductor $(Ca_4Al_2O_{6-y})(Fe_2As_2)$: ^{75}As nuclear quadrupole resonance study," Phys. Rev. Lett. **107**, 047002 (2011).

[30] K. Yamamoto *et al.*, "Antiferromagnetic order and superconductivity in $Sr_4(Mg_{0.5-x}Ti_{0.5+x})_2O_6Fe_2As_2$ with electron

doping: ^{75}As-NMR study," J. Phys. Soc. Jpn. **81**, 053702 (2012).
[31] J. Munevar *et al.*, "Static magnetic order of $Sr_4A_2O_6Fe_2As_2$ (A = Sc and V) revealed by Mössbauer and muon spin relaxation spectroscopies," Phys. Rev. B **84**, 024527 (2011).
[32] S. Backes *et al.* "Microscopic nature of correlations in multiorbital AFe_2As_2 (A = K, Rb, Cs): Hund's coupling versus Coulomb repulsion," Phys. Rev. B **92**, 195128 (2015).
[33] M. Yi *et al.* "Symmetry-breaking orbital anisotropy observed for detwinned $Ba(Fe_{1-x}Co_x)_2As_2$ above the spin density wave transition," Proc. Natl. Acad. Sci. USA **108**, 6878 (2011).
[34] K. Kitagawa *et al.*, "Stripe antiferromagnetic correlations in $LaFeAsO_{1-x}F_x$ probed by ^{75}As NMR," Phys. Rev. B **81**, 212502 (2010).
[35] M. Hirano *et al.*, "Potential antiferromagnetic fluctuations in hole-doped iron-pnictide superconductor $Ba_{1-x}K_xFe_2As_2$ studied by ^{75}As nuclear magnetic resonancemeasurement," J. Phys. Soc. Jpn. **81**, 054704 (2012).
[36] J. Cui *et al.*, "Antiferromagnetic spin correlations and pseudogaplike behavior in $Ca(Fe_{1-x}Co_x)_2As_2$ studied by ^{75}As nuclear magnetic resonance and anisotropic resistivity," Phys. Rev. B **92**, 184504 (2015).
[37] L. Ortenzi, *et al.*, "Accounting for spin fluctuations beyond local spin density approximation in the density functional theory," Phys. Rev. B **86**, 064437 (2012).
